# Parsing (malicious) pleasures: schadenfreude and gloating at others’ adversity

**DOI:** 10.3389/fpsyg.2015.00201

**Published:** 2015-02-26

**Authors:** Colin Wayne Leach, Russell Spears, Antony S. R. Manstead

**Affiliations:** ^1^Department of Psychology, University of ConnecticutStorrs, CT, USA; ^2^Social Psychology, Faculty of Behavioral and Social Sciences, University of GroningenGroningen, Netherlands; ^3^School of Psychology, Cardiff UniversityCardiff, UK

**Keywords:** schadenfreude, gloating, pride, joy, satisfaction, emotion, group, competition

## Abstract

We offer the first empirical comparison of the pleasure in seeing (i.e., schadenfreude) and in causing (i.e., gloating) others’ adversity. In Study 1, we asked participants to recall and report on an (individual or group) episode of pleasure that conformed to our formal definition of schadenfreude, gloating, pride, or joy, without reference to an emotion word. Schadenfreude and gloating were distinct in the situational features of the episode, participants’ appraisals of it, and their expressions of pleasure (e.g., smiling, boasting). In Study 2, we had participants imagine being in an (individual or group) emotion episode designed to fit our conceptualization of schadenfreude or gloating. Individual and group versions of the emotions did not differ much in either study. However, the two pleasures differed greatly in their situational features, appraisals, experience, and expression. This parsing of the particular pleasures of schadenfreude and gloating brings nuance to the study of (malicious) pleasure, which tends to be less finely conceptualized and examined than displeasure despite its importance to social relations.

## INTRODUCTION

To see others suffer does one good, to make others suffer even more: this is a hard saying but an ancient, mighty, human, all-too-human, principle to which even the apes might subscribe

(Nietzsche, 1887/1967, p. 67).

Nietzsche had a less than generous view of human nature. He argued that other people’s adversity was an important source of pleasure. However, in his view, passively observing others’ adversity provides a different pleasure than actively causing others’ adversity oneself by directly defeating them in competition. Was Nietzsche correct? We offer the first empirical comparison of the pleasure in passively observing (i.e., schadenfreude) and in actively causing (i.e., gloating) others’ adversity.

Because emotion words can be imprecise descriptions of emotion concepts, and because schadenfreude and gloating are lesser-known emotion words, in a first study we asked participants to recall and report an episode of a “positive feeling” that conformed to our conceptualization of schadenfreude or gloating (as well as pride or joy). Thus, we made no reference to emotion words in our prompts. We examined the situational features of the episode, participants’ appraisals of it, and their expression of pleasure (e.g., smiling, boasting) about the episode. In a second study, we parsed more finely the experience and the expression of schadenfreude and gloating by having participants imagine being in a particular episode of our design. Because previous research on schadenfreude has focused on *either* individual or group instances, our two studies compared such instances of schadenfreude and gloating. Our parsing of the particular pleasures of schadenfreude and gloating seeks to bring the sort of nuance routinely applied to dysphoric emotions to the less finely conceptualized and examined euphoric emotions. As important as this nuance is conceptually, it is also important to understand the ways in which schadenfreude and gloating may be dramatically different orientations to the adversity of other people with distinct implications for social relations (Leach et al., 2003).

## PARSING (MALICIOUS) PLEASURES

Although common decency may limit malicious pleasure, it is clear that people do sometimes enjoy the adversity suffered by other individuals (e.g., Smith et al., 1996; van Dijk et al., 2005) and out-groups (e.g., Leach et al., 2003; Combs et al., 2009). Popular discussions use the term schadenfreude to describe many malicious pleasures, including pleasure at witnessing others’ foibles on “reality TV”; pleasure at a celebrity’s narcissistic self-destruction through pills, spills, or untoward thrills; and pleasure at seeing those of questionable virtue punished or otherwise given their comeuppance (for discussions, see Kristjánsson, 2006, Chap. 3; Lee, 2008). At least since Heider’s (1958, Chap. 11) influential analysis, psychologists have paralleled popular discussions and used the term schadenfreude to describe any pleasure at any adversity that befalls another party (for discussions, see Feather, 2006; Koenig, 2009; Leach et al., 2014). This broad definition of schadenfreude is also used in philosophy (e.g., Portmann, 2000; Ben-Ze’ev, 2001; but see Kristjánsson, 2006) and in a variety of other disciplines (for a review, see van Dijk and Ouwerkerk, 2014). This use of schadenfreude to describe any and all pleasure at another’s adversity is part of a more general trend in the study of positively experienced emotion. Generally speaking, pleasures are conceptualized and examined less finely than displeasures (Averill, 1980; de Rivera et al., 1989; more generally, see Frijda, 1986; Shaver et al., 1987; Ortony et al., 1988; Lazarus, 1991).

It seems clear, however, that all pleasure at adversity is not the same. Misfortune, direct defeat, deserved failure, and comeuppance are very different types of adversity. Thus, it seems reasonable to expect that the pleasure experienced at each of these adversities is different. Indeed, pleasure at a rival’s misfortune is about something very different than pleasure at defeating a rival oneself or at seeing a rival deservedly punished. One important way in which emotion concepts can be differentiated conceptually is to specify what the experience of pleasure or displeasure is about (Frijda, 1986; Lazarus, 1991; Solomon, 1993, Chap. 5). For example, pride works well as an emotion concept because it is conceptualized as pleasure about the particular advantage of a deserved success that is distinct from the pleasure of joy or love (Frijda, 1986; Ortony et al., 1988; Lazarus, 1991).

Defining schadenfreude as (any) pleasure at (any) adversity suffered by another party is akin to defining pride as (any) pleasure at (any) good fortune for the self. Such a general definition undermines the value of specific emotion concepts. For this reason alone, schadenfreude should be defined as a specific pleasure about a particular kind of adversity that can be conceptually and empirically differentiated from other pleasure at adversity (such as gloating), in terms of its situational features, typical appraisals, and the quantity and quality of the experience and expression of pleasure. More practically, a finer conceptualization of pleasure at adversity can clarify how malicious emotions like schadenfreude and gloating constitute different ways of relating to those suffering adversity. Emotions can be conceptualized as relational states, in the sense that they both reflect and arguably constitute social relationships. Lazarus (1991) argued that emotions are characterized by ‘core relational themes’ that capture the relational meaning of an encounter for the individual. Although Lazarus’ primary focus was on the person–environment relationship, other people are key features of the environment in many emotional episodes. The result is that some of Lazarus’ core relational themes (e.g., those for guilt, pride, envy, jealousy, love, and compassion) are social-relational in nature. Other theorists (e.g., de Rivera, 1984; Parkinson, 1996; Tiedens and Leach, 2004; Parkinson et al., 2005) have adopted a more explicitly social-relational view of emotions, arguing that emotions both reflect and shape ongoing social relationships. Considered from this perspective, it should be possible to distinguish schadenfreude and gloating in terms of the position of the self relative to the other party. For example, the wish to flaunt the pleasure of gloating puts the self above the defeated party, who is belittled.

### SCHADENFREUDE vs. GLOATING

Nietzsche (1887/1967) described schadenfreude as pleasure at the passive observation of another party’s misfortune. Because the observer does nothing to “earn” schadenfreude, Nietzsche viewed the pleasure of schadenfreude as lesser than pleasure that is actively earned. He also suggested that those experiencing schadenfreude are less empowered than those who actively “make others suffer” by directly defeating them in competition. Pleasure in actively and directly causing a rival’s adversity may be referred to as *gloating*, especially when it is experienced as an empowered state of superiority that is lorded over the defeated rival (Ortony et al., 1988). Like Nietzsche, we believe that the emotion concept of schadenfreude should describe a particular pleasure at adversity that is distinguishable from other pleasure (e.g., pride and joy). We also believe that schadenfreude should describe a particular pleasure at another’s adversity that is distinguishable from other pleasure at another’s adversity (e.g., gloating). More specifically, the malicious pleasures of schadenfreude and gloating should be *experienced* differently, with schadenfreude less pleasurable, less empowering, and more passive and indirect than gloating. Schadenfreude and gloating should also be *expressed* differently, because gloating should be boastful and triumphant in nature and schadenfreude should be more furtive. The experience and expression of schadenfreude and gloating should be corroborated by the quite different ways that the two malicious pleasures position the self in social relations. Whereas gloating is an experience and expression of superiority over others, the muted pleasure of schadenfreude is based in passivity and concerns about inferiority and powerlessness. Thus, the distinctions between schadenfreude and gloating can be conceptualized in terms of the (1) features of the event, (2) appraisals of the event, (3) experience of pleasure, and (4) expression of pleasure. These distinctions are shown in **Table 1**.

**Table 1:** Conceptual distinctions between schadenfreude and gloating.

	Schadenfreude	Gloating
**Features of event**
Competition	Indirect, moderate	Direct, high
Comparison	Moderate	Moderate
Self-benefit	Indirect, moderate	Direct, high
Vantage point	(passive) Observer	Actor
**Appraisals**
Agency	External	Internal
Power	Low	Moderate to high
Status	Moderate	High
Performance	Moderate	High
**Experience**
Degree of pleasure	Moderate	High
Activity	Moderate	High
Elevated		High
Triumphant		High
Emboldened		High
**Expression**
	Suppressed	Expressed
	Private	Public
Smiling	Moderate (suppressed)	High
Celebration/glee	Low to moderate	High
Flaunting/boasting	Low to moderate	High

We expect that the features of the event that precipitates schadenfreude will be quite different than those of the event that precipitates gloating. As shown in **Table 1**, we follow Nietzsche in expecting that schadenfreude is characterized by a moderate level of indirect competition, in contrast to the high level of direct competition that should characterize gloating. Because of the direct competition, there should be more direct material benefit to the self in gloating events; the gain in schadenfreude is more psychological (see also Leach et al., 2003; Leach and Spears, 2009).

A central feature of schadenfreude is that one is a passive observer of the event rather than an active actor (Ben-Ze’ev, 2001, Chap. 12; Leach et al., 2003). Thus, schadenfreude and gloating should differ dramatically in appraisals of agency. Whereas something or someone other than the self should be appraised as the agent of the other’s adversity in schadenfreude (see also Ben-Ze’ev, 2001, Chap. 12; Leach et al., 2014), the self should be appraised as the agent in gloating (see also Ortony et al., 1988). And, in comparison to schadenfreude, gloating should be characterized by greater appraisals of the self as having power and status, and performing successfully (see Nietzsche, 1887/1967; Ortony et al., 1988).

As Nietzsche (1887/1967) argued, the experience of gloating should be more pleasurable than schadenfreude. We also expect the experience of the two pleasures to differ in quality. In comparison to passive schadenfreude, the phenomenological experience of gloating should be embodied as a state of physical activation and arousal. Gloating should also be embodied as a greater state of physical elevation, as people should feel “10 feet tall” and “on top of the world” when they defeat a rival in this way. This elevated phenomenology is consistent with the appraisals of power and status that characterize gloating and schadenfreude (for a general discussion, see Schubert, 2005). Thus, those experiencing gloating should also feel more triumphant (i.e., victorious, proud) and emboldened (i.e., bold, fearless) than those experiencing schadenfreude.

As shown in **Table 1**, we also expect the expression of pleasure to be quite different in schadenfreude and gloating. A central part of gloating is to express openly one’s pleasure at defeating a rival (see also Ortony et al., 1988). This should include smiling and may include celebrating and expressing glee. It may even include the more malicious expressions of boasting and flaunting one’s pleasure in front of the defeated rival. Such expressions are less characteristic of schadenfreude. In fact, the passive and indirect nature of schadenfreude, and its muted pleasure, suggests that it may be furtive in expression (see Leach et al., 2003). As a more private pleasure, those experiencing schadenfreude seem likely to suppress their public expression of pleasure. They may hide a smile, in part because they feel bad about taking “unearned” pleasure in another’s adversity.

### INDIVIDUAL vs. GROUP-BASED EMOTION

Since Smith’s (1993) call for greater attention to emotions about group and inter-group events, much research has been conducted. However, only a few papers have examined schadenfreude about group adversity (Leach et al., 2003; Leach and Spears, 2008, 2009; Combs et al., 2009) and no papers have examined gloating about groups. In addition, none of the work on schadenfreude, and little of the work on other emotions, has directly compared emotions about individual and group events (for reviews, see Parkinson et al., 2005; Iyer and Leach, 2008). Thus, we thought it important to examine both individual and group schadenfreude and gloating. As long as individual and group events are equally relevant to the corresponding level of self, individual and group-based emotions should have similar signatures (Iyer and Leach, 2008). Indeed, if group-based emotion is genuine emotion, it should operate in ways parallel to individual emotion. Where individual and group emotion are most likely to differ is in those aspects of emotion most affected by social sharing with others, which may be more likely within groups having a shared experience (e.g., watching the Olympics together with co-nationals; for discussions, see Tiedens and Leach, 2004; Parkinson et al., 2005).

## STUDY 1

Our main purpose was to compare the appraisals and expressions characteristic of schadenfreude and gloating, about both individual and group events. However, we also thought it important to compare these two malicious pleasures to more benign pleasures. Thus, we also compared schadenfreude and gloating to two widely discussed pleasures – pride and joy.

We used a variation of emotion recall methodology. The typical technique would involve asking participants to recall and report on a recent episode of “schadenfreude,” “gloating,” “pride,” or “joy.” However, this technique makes the potentially problematic assumption that participants have a clear and consensual understanding of the emotion words with which they are presented (Wierzbicka, 1992). This assumption is clearly wrong in the case of schadenfreude, a word that has only recently been imported into English. Although the emotion words gloating, pride, and joy are less obscure than schadenfreude, it also seemed unwise to assume that participants would share our formal definitions of these emotion concepts. In fact, it is clear that emotion words operate in everyday language as “fuzzy concepts” whose meaning is variable (Shaver et al., 1987; Ortony et al., 1988; Wierzbicka, 1992). Thus, we eschewed the use of emotion words and instead asked participants to recall an episode that we described in terms consistent with our definitions of schadenfreude, gloating, pride, and joy. This approach focuses on the idea that an emotion can be clearly defined by what it is about (Solomon, 1993). As such, our method is freer of individual and cultural particularities than methods that ask participants to recall an experience labeled with an ambiguous emotion word (Wierzbicka, 1992).

### METHOD

#### Participants

One hundred and nine (91 women, 18 men) students at a British university participated for partial course credit^1^. They identified as English (53), British (24), Welsh (13), Irish (2), Scottish (1), or “other” (16). Participants ranged in age from 18 to 33, *M* = 20.5, SD = 2.46. Ethical approval for both this study and Study 2 (below), was obtained in advance from the departmental research ethics committee, conforming to American Psychological Association and British Psychological Society guidelines (e.g., all participants gave informed consent, were advised that they could withdraw at any time without penalty, and were fully debriefed at the end of their participation).

#### Design

This study employed a 4 (Emotion recalled: schadenfreude, gloating, pride, joy) × 2 (Level: individual vs. group-based emotion) × 2 (Order: individual vs. group first) design. Level and order were within-participants factors. Emotion recalled was a between-participants factor. There were between 26 (gloating) and 28 (schadenfreude, pride) participants in each condition. Because order had no statistically significant effects, it is not discussed further.

Given the complexity of our design, it was necessary to treat some factors as within-participant. Because we expected the distinction between individual and group-based emotion to be subtle we chose to maximize statistical power for this comparison by treating it as a within-participants factor. Because we expected the distinctions between the four pleasures to be larger, statistical power should be adequate with emotion as a between-participants factor. It was also advantageous to treat emotion as a between-participants factor because this would obscure our interest in comparing the four pleasures from participants. Having each participant report on all four emotions would have likely made our research interests obvious and would have likely led to demand characteristics that would distort results. We expected participants to be less reactive to being asked about both individual and group-based examples of a given emotion.

#### Procedure

In the first part of the study, participants were asked to “Think back to a specific time in your life when you had a positive feeling… (emphasis in original).” They were then asked to “give as much detail as you can about how you felt at this time and try to say what it was *precisely* that made you come to feel good in the way that you did.” In each condition, the positive feeling was described in a way consistent with our conceptualization of schadenfreude, gloating, pride, or joy. Thus, in the schadenfreude condition, participants were asked about “a positive feeling resulting from someone else (a group to which you did not belong) suffering a defeat, failure, or other negative outcome […] even though you (your group) played no role in causing this outcome.” In the gloating condition, we asked about “positive feelings resulting from (a group to which you belonged) triumphing over, or defeating, another person (group).” In the pride condition, we asked about “strong positive feelings (as a member of a group,) resulting from an individual (group) achievement.” And, in the joy condition, we asked about a “sudden and intense positive feeling (as a group member), resulting from something pleasurable happening.”

#### Equivalence checks

**FIGURE 1:**
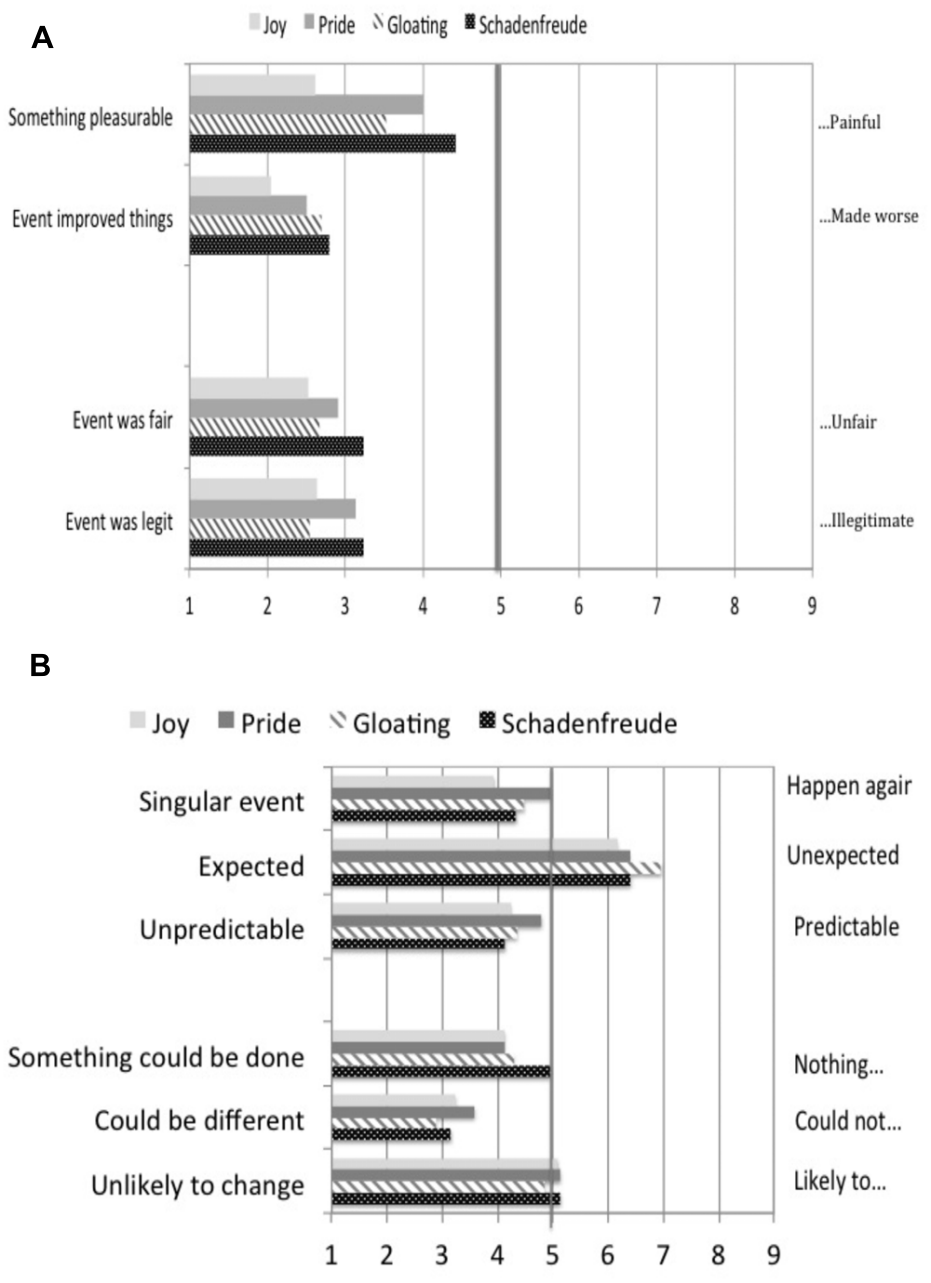
**(A)** Equivalence checks: perceived pleasure and fairness of emotion episodes, Study 1. **(B)** Equivalence checks: perceived predictability and changeability of emotion episodes, Study 1.

To be sure that each emotion condition was equivalent, we included a series of checks based in items used by Roseman et al. (1990). All items asked participants to indicate to what degree “my feelings were caused by…” Responses were presented in a 9-point bi-polar scale anchored by statements at each end (see **Figures 1A,B**).

The perceived pleasure of the emotion episodes was measured with two questions that asked to what degree participants’ feelings were caused by “believing that what happened improved things” (1) or “…made things worse” (9) and “wanting to get or keep something pleasurable” (1) or “wanting to get rid of or avoid something painful” (9). The perceived fairness of the episode was measured with questions that asked whether the episode “…was fair” (1) or “was unfair” (9) and “…was legitimate” (1) or “was illegitimate” (9).

The perceived predictability of the episodes was measured with questions that asked whether “feelings were caused by […] thinking that I was unable…” (1) or “…able to predict what was going to happen” (9); “perceiving something as expected” (1) or “…as unexpected” (9); and “…what happened was a one-off event” (1) or “…likely to happen again” (9). The perceived changeability of the emotion episodes was measured with three questions that asked to what degree participants’ feelings were caused by thinking that what happened “was due to a situation that was unlikely to change” (1) or “…likely to change” (9); “…what happened could have turned out differently” (1) or “…could *not* have turned out differently (9); and “…something could be done about this situation” (1) or “…nothing could be done” (9).

#### Appraisals

Based on Roseman et al. (1990), we assessed a series of appraisals by asking participants to indicate to what degree “my feelings were caused by…” Responses were presented in a 9-point bi-polar scale anchored by statements at each end.

***Agency*.** The agency in the precipitating event was measured with three questions that assessed to what degree participants’ feelings were caused by thinking that “…what happened was not at all due to me” (1) or “…was very much due to me” (9); “…what happened was not at all due to someone else” (1) or “…was very much due to someone else” (9); and “…I had a central role in what happened” (1) or “…I was an observer of what happened” (9).

***Power*.** The participants’ appraisal of their power in the precipitating event was measured with questions stating that “I had the resources to affect what happened” (1) or “I did not have the resources…” (9); and “…I had the power to change what happened” (1) or “…I was powerless…” (9).

***Performance*.** Participants’ appraisal of their performance in the event was assessed with two questions asking if their feelings were caused by thinking that “…I had failed” (1) or “…I had succeeded” (9); and “…I was unsuccessful” (1) or “…I was successful” (9).

***Status*.** Participants’ appraisal of their status in the event was assessed with two questions asking if their feelings were caused by thinking that “…I was worse than the other person” (1) or “I was better…” (9); and “…I was inferior” (1) or “…I was superior…” (9).

#### Actions

In a series of questions, we asked participants “to indicate the extent to which” they “actually engaged” in the following behavior during the emotion episode: “I smiled,” “I kept the feeling of pleasure to myself,” “I celebrated,” “I “freely expressed my glee,” “I flaunted my feelings of pleasure” and “I boasted about what happened.” All items were presented with a 9-point response scale that ranged from *not at all* (1) to *very much so* (9).

### RESULTS

#### Coding of emotion narratives

Two coders examined the emotion narratives for specific features of the event and explicitly stated appraisals of agency. The coders agreed 81% of the time. Disagreements were settled by discussion. Results are presented in **Table 2**. In a pairwise comparison, gloating involved more direct competition than schadenfreude, χ^2^(1) = 17.77, *p* < 0.001, as well as more direct competition than joy and pride, both *p* < 0.001^2^. Also as expected, gloating involved more direct benefit than schadenfreude, χ^2^(1) = 19.49, *p* < 0.001, as well as more than joy, χ^2^(1) = 7.28, *p* = 0.007, and pride, χ^2^(1) = 13.14, *p* < 0.001. Although the gloating and schadenfreude conditions did not differ from each other in the degree of direct comparison, χ^2^(1) = 0.154, *p* = 0.690, gloating and schadenfreude involved greater comparison than joy or pride, all *p* < 0.001. Lastly, schadenfreude was characterized by the least self-agency, χ^2^(3) = 12.00, *p* = 0.007. Consistent with this, others [χ^2^(3) = 13.24, *p* = 0.001], and third parties [χ^2^(3) = 39.27, *p* < 0.001] were more frequently said to be agents in narratives of schadenfreude.

**Table 2:** Quantitative coding of event features and appraisals in emotion narratives, Study 1.

	Emotion narratives			
Coding categories	Joy	Pride	Gloating	Schaden-freude
Direct competition^a^	23%	15%	**67%**	26%
χ^2^(3) = 38.25, *p* < 0.001				
Direct benefit from misfortune^a^	39%	30%	**56%**	23%
χ^2^(3) = 22.75, *p* < 0.001				
Direct comparison^a^	08%	09%	**41%**	**37%**
χ^2^(3) = 27.04, *p* < 0.001				
Agency^b^				
Self (individual or group)	85%	96%	90%	**39%**
χ^2^(3) = 12.00, *p* = 0.007			
Other (individual or group)	08%	00%	04%	**20%**
χ^2^(3) = 13.24, *p* = 0.001			
Third party (individual or group)	00%	00%	00%	**30%**
χ^2^(3) = 39.27, *p* < 0.001			
Luck/happenstance^c^	06%	00%	06%	**11%**

*Frequencies found to most differ from others in the same row are shown in bold.*

*^a^Coded as either “not mentioned” (0) or “mentioned” (1). ^b^This Chi-square uses Yates’s correction for continuity to improve the accuracy of tests that include cells with small or zero values (see Preacher, 2001). ^c^Small frequencies in three conditions precluded a statistical test.*

#### Equivalence checks

These single questions were analyzed individually in a mixed-model analysis of variance (ANOVA). Because of the numerous statistical tests conducted, it is important to attend to the $\eta_{p}^{2}$ index of effect size as well as the actual *p*-value of “statistical significance.” Larger effect sizes and smaller *p*-values offer more secure statistical inference in light of the number of tests we report. Results are shown in **Figure 1A**.

There was a significant effect of emotion condition on the perception that the event was about “wanting to get or keep something pleasurable,” *F*(3,108) = 5.73, *p* = 0.001, $\eta_{p}^{2}$ = 0.144. However, pairwise comparisons showed that the pride, gloating, and schadenfreude conditions were seen as equally pleasurable (all *p*s > 0.10). There was no effect of emotion condition on the perception that the event “improved things,” *F*(3,108) = 1.70, *p =* 0.171, $\eta_{p}^{2}$ = 0.046. There were no significant main effects or interactions involving individual vs. group emotion, all *p*s > 0.092.

As shown in the bottom half of **Figure 1A**, the precipitating event was seen as equally “fair,” *F*(3,108) = 1.13, *p* = 0.342, $\eta_{p}^{2}$ = 0.031. There was no significant main effect or interaction involving individual vs. group emotion, all *p*s > 0.260. The event was also seen as equally “legitimate” across the four emotion conditions, *F*(3,105) = 1.42, *p* = 0.242, $\eta_{p}^{2}$ = 0.039. However, the group emotions (*M* = 2.64, SE = 0.148) were appraised as more legitimate than the individual emotions (*M* = 3.12, SE = 0.210), *F*(3,108) = 4.88, *p* = 0.029, $\eta_{p}^{2}$ = 0.044. There was no two-way interaction, *F*(3,108) = 0.236, *p* = 0.718, $\eta_{p}^{2}$ = 0.007.

It can be seen in the top half of **Figure 1B** that the precipitating events were judged to be equally predictable across the four emotion conditions, all *p*s > 0.250, all $\eta_{p}^{2}$ < 0.038. However, the individual emotion events (*M* = 6.88, SE = 0.203) were seen as more unexpected than those for group emotions (*M* = 6.09, SE = 0.204), *F*(3,108) = 10.49, *p* = 0.002, $\eta_{p}^{2}$ = 0.091. The precipitating events were seen as equally changeable, all *p* > 0.214, all $\eta_{p}^{2}$ < 0.042. There were no significant main effects of individual vs. group emotion, all *p* > 0.482, all $\eta_{p}^{2}$ < 0.005. Together, these results established that the four emotions were equivalent in these numerous ways, ruling out these appraisals as alternative explanations of our results.

#### Appraisals

These single questions were again analyzed individually in a mixed-model ANOVA.

**FIGURE 2:**
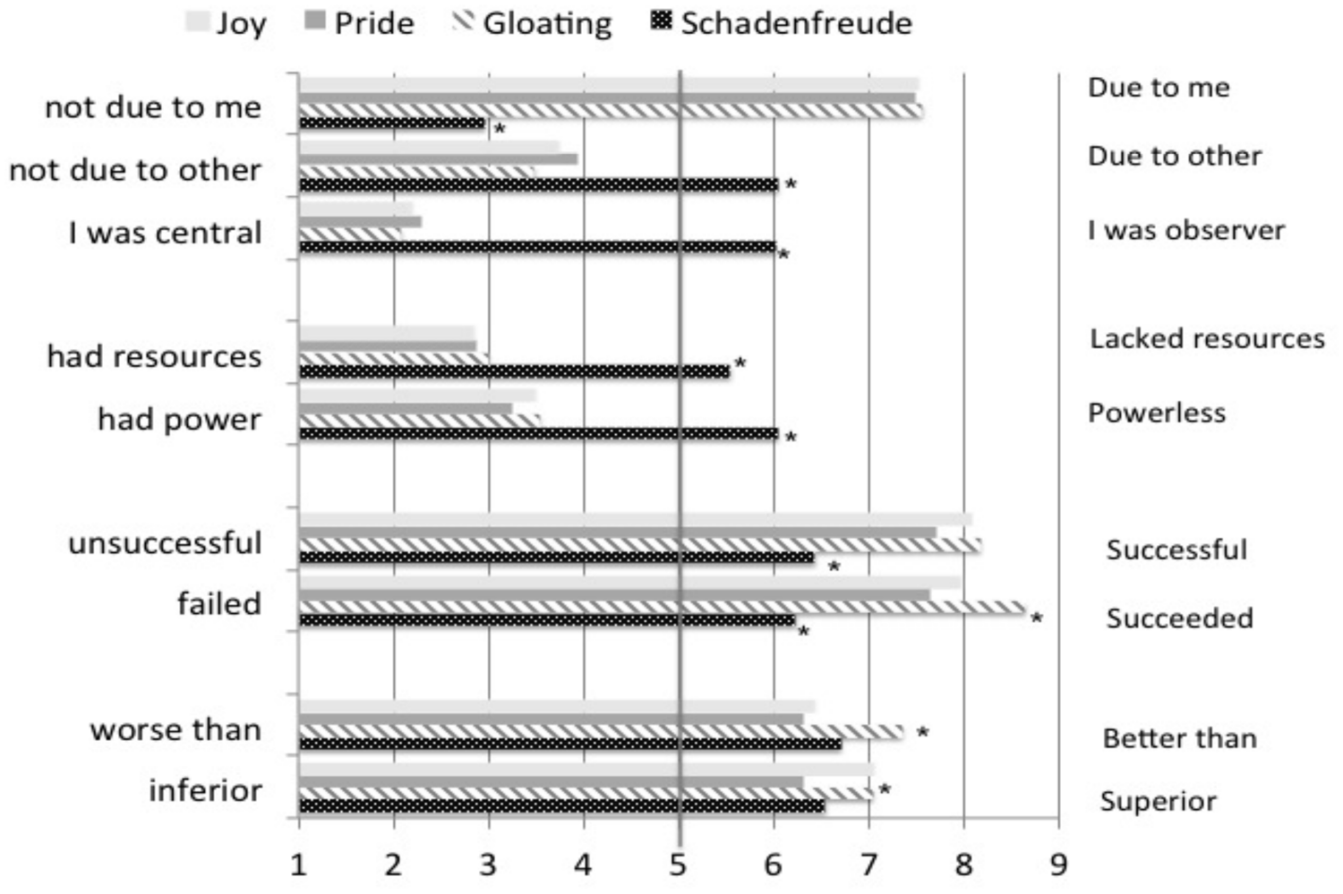
**Appraisals of agency, power, performance, and status, Study 1.** Asterisks show that the emotion condition in question differed significantly from one or more of the other emotion conditions.

***Agency*.** As shown in first section of **Figure 2**, participant’s appraisal that their feeling was caused by something “due to me” was affected by the emotion condition, *F*(3,104) = 60.46, *p* < 0.001, $\eta_{p}^{2}$ = 0.636, with the lowest endorsement in the schadenfreude condition, all pairwise comparisons *p* < 0.001. Individual vs. group emotion was not significant, both *p*s > 0.339. The appraisal that what happened was “due to someone else” was also affected by the emotion condition, *F*(3,105) = 12.89, *p* < 0.001, $\eta_{p}^{2}$ = 0.269, with the highest endorsement in the schadenfreude condition (all pairwise *p*s < 0.001). The appraisal that the event was “due to someone else” was also higher in the group (*M* = 4.73, SE = 0.219) than the individual (*M* = 3.87, SE = 0.232) emotion conditions, *F*(3,105) = 8.02, *p* = 0.006, $\eta_{p}^{2}$ = 0.071. Lastly, there was only an effect of emotion condition on the appraisal that the participant was an observer of what happened, *F*(3,105) = 41.18, *p* < 0.001, $\eta_{p}^{2}$ = 0.541, with the highest endorsement in the schadenfreude condition (all *p*s < 0.001). Individual vs. group emotion was not significant, both *p*s > 0.241.

***Power*.** As shown in second section of **Figure 2**, participants’ appraisal that they did “not have the resources to affect what happened” was affected by emotion condition, *F*(3,104) = 16.48, *p* < 0.001, $\eta_{p}^{2}$ = 0.322, with endorsement highest in the schadenfreude condition (all *p*s < 0.001). Individual vs. group emotion was not significant, both *p* > 0.074. In addition, the appraisal that they were “powerless to change what happened” was significantly affected by emotion condition, *F*(3,105) = 14.06, *p* < 0.001, $\eta_{p}^{2}$ = 0.287, with endorsement highest in the schadenfreude condition (all *p* < 0.001). Appraisals of power were higher in the individual (*M* = 4.34, SE = 0.214) than group (*M* = 3.82, SE = 0.204) emotion conditions, *F*(3,105) = 5.46, *p* = 0.021, $\eta_{p}^{2}$ = 0.049.

***Performance*.** As shown in the third section of **Figure 2**, participants’ appraisal that they were “successful” was affected by emotion condition, *F*(3,104) = 12.24, *p* < 0.001, $\eta_{p}^{2}$ = 0.255, with endorsement lowest in the schadenfreude condition (all *p* < 0.001). Also, participants’ appraisal that they “succeeded” rather than “failed” was only significantly affected by emotion condition, *F*(3,104) = 13.09, *p* < 0.001, $\eta_{p}^{2}$ = 0.269, with the schadenfreude condition lower than all others (all *p*s < 0.001). Individual vs. group emotion had no significant main or interaction effect.

***Status.*** As shown in the final section of **Figure 2**, participants tended to appraise themselves as having the highest status in the gloating condition, although these effects were small and statistically marginal. Specifically, participants’ appraisal that they were better than the other person was marginally affected by emotion condition, *F*(3,105) = 2.59, *p* = 0.057, $\eta_{p}^{2}$ = 0.069. Pairwise comparisons showed the gloating condition to be significantly higher than the joy (*p* = 0.025) and pride (*p* = 0.012) conditions, but not the schadenfreude condition (*p* = 0.109). Surprisingly, there was also an interaction between emotion condition and individual vs. group emotion, *F*(3,105) = 4.65, *p* = 0.004, $\eta_{p}^{2}$ = 0.117. The pattern of results was inconsistent across emotion conditions. Participants’ appraisal that they were superior was marginally affected by emotion condition, *F*(3,104) = 2.21, *p* = 0.091, $\eta_{p}^{2}$ = 0.060. Pairwise comparisons showed the gloating condition to be significantly higher than the pride (*p* = 0.040) condition, but not the joy (*p* = 0.997) or schadenfreude (*p* = 0.153) conditions.

#### Actions

These single questions were analyzed individually in mixed-model ANOVAs. Means are shown in **Table 3**. The least smiling was reported in the schadenfreude condition, all *p*s < 0.026. In addition, the schadenfreude condition yielded the least celebration, all *p*s ≤ 0.001. Also, glee was more freely expressed in the gloating than in the schadenfreude condition, *p* = 0.005, and pleasure was flaunted more in the gloating than in the schadenfreude condition, *p* = 0.033. Participants boasted only marginally more in the gloating than in the pride (*p* = 0.076) and schadenfreude (*p* = 0.100) conditions.

**Table 3:** Reported expression of pleasure by emotion condition, Study 1.

	Emotion narratives			
	Joy	Pride	Gloating	Schadenfreude
	*M* (SE)	*M* (SE)	*M* (SE)	*M* (SE)
Smiled	8.19 (0.263)	7.84 (0.259)	7.69 (0.268)	**6.87** (0.246)
*F*(3,105) = 4.90, *p* = 0.003, $\eta_{p}^{2}$ = 0.120				
Kept pleasure to myself	**2.87** (0.335)	3.64 (0.329)	3.73 (0.342)	4.02 (0.329)
*F*(3,105) = 2.16, *p* = 0.097, $\eta_{p}^{2}$ = 0.058				
Celebrated	7.32 (0.361)	6.66 (0.355)	6.54 (0.368)	**4.89** (0.337)
*F*(3,105) = 8.96, *p* < 0.001, $\eta_{p}^{2}$ = 0.199				
Expressed my glee	6.69 (0.319)	5.73 (0.313)	5.87 (0.325)	**4.61** (0.298)
*F*(3,105) = 7.72, *p* < 0.001, $\eta_{p}^{2}$ = 0.177				
Flaunted my pleasure	**6.07** (0.346)	4.82 (0.340)	5.19 (0.352)	4.16 (0.323)
*F*(3,105) = 5.65, *p* < 0.001, $\eta_{p}^{2}$ = 0.136				
Boasted	**5.65** (0.377)	4.41 (0.370)	5.37 (0.384)	4.50 (0.352)
*F*(3,108) = 2.78, *p* = 0.044, $\eta_{p}^{2}$ = 0.072				

*Means found to most differ from others in the same row are shown in bold.*

### DISCUSSION

Study 1 generally confirmed our predictions regarding the signature of schadenfreude. Thus, schadenfreude was characterized by appraisals that others, rather than the self, were the agent of the precipitating event. Schadenfreude was also unique in being experienced as a state of lower power and performance. Unlike, gloating, joy, and pride, the pleasure in schadenfreude was expressed somewhat furtively; there was less reported smiling and less glee, boasting, and flaunting of participants’ pleasure.

As well as being distinct from schadenfreude, gloating tended to be as pleasurable as joy – the most pleasurable emotion we examined. Gloating and joy also tended to be about equal in openly expressing pleasure. This further confirms the intense pleasure of “making others suffer” by defeating them in direct competition. Importantly, gloating was also characterized by greater boasting than was pride. Although we performed a good number of statistical tests to examine every specific appraisals, experiences, and expressions of the four pleasures, observed differences tended to be consistent, highly “statistically significant,” and moderate to large in size. This gives us confidence that these differences are unlikely to be due to the greater chance introduced by the number of statistical tests we conducted.

Importantly, the equivalence checks showed that the emotion conditions were equivalent in a number of important ways. The gloating, schadenfreude, joy, and pride episodes were seen as equally fair and legitimate, and as equally predictable and changeable. Thus, there was little difference in what participants had “at stake” in the schadenfreude and gloating situations, or in the individual or group situations. This rules out the alternative explanation that the schadenfreude and gloating episodes differed so much because the schadenfreude episode was less important to participants than the gloating episode. The possibility that the observed differences between schadenfreude and gloating reflect a response bias that encouraged less expression of everything related to schadenfreude was also ruled out. As expected, schadenfreude was rated higher on a number of appraisals (e.g., powerlessness, other-agency).

The present results are also notable for the consistent pattern of parallel effects across the individual and group instances of the emotions. The manipulation of individual vs. group emotion rarely had effects on the experience or the expression of the pleasures. However, as expected, the group-based pleasures were occasionally expressed more openly. Importantly, the individual and group instances of schadenfreude and gloating did not tend to differ from each other. This demonstrates the generalizability of the findings across individual and group instances.

## STUDY 2

In Study 2 we aimed to corroborate and extend Study 1 in several ways. First, we focused more precisely on the differences between schadenfreude and gloating by examining only these two emotions. Second, we wished to complement the emotion recall procedure of Study 1, in which participants generated their own, somewhat idiosyncratic, episodes of emotion, by using a vignette method in which participants were asked to imagine a particular episode of pleasure that conformed to our conceptualization of schadenfreude or gloating. Third, we aimed to corroborate our findings regarding the similarity between individual and group schadenfreude and gloating using a between-participants design. This complements the within-participants design in Study 1, which may have encouraged participants to respond in similar ways in individual and group instances of the emotions. Fourth, we extended our measures beyond those used in Study 1 to make more elaborate assessments of the ways in which the pleasures differ in experience (i.e., form of pleasure, physical activity, elevated phenomenology) and expression (gloating, smiling, celebrating, flaunting, suppressing).

### METHOD

#### Participants and design

Participants were 125 students (25 men and 100 women) at the same university as Study 1. They were rewarded either with course credit or payment of £3. Participants’ ages ranged from 17 to 45, *M* = 21, SD = 4.0. Participants were randomly assigned to one of the four experimental conditions in a 2 (individual vs. group emotion) × 2 (schadenfreude vs. gloating) between-participants design.

#### Procedure

After providing consent and completing some demographic questions, participants were asked to vividly imagine taking part in an event. In the interpersonal condition, the participant was asked to imagine that s/he was an individual competing against a rival for a place on the university’s field hockey team. In the inter-group condition, the participant was asked to imagine that s/he was a member of the university hockey team competing against rival universities. A second section of the vignette then offered the participants an opportunity for gloating or schadenfreude. The gloating opportunity was presented by having participants imagine succeeding against their rival. The schadenfreude opportunity was presented by having their rival fail against a third party.

#### Measures

Measures included checks on the equivalence of the vignettes, four kinds of emotion experience and five kinds of emotion expression.

***Equivalence checks*.** Participants were asked to indicate to what degree they felt “a sense of rivalry,” “hostile” toward their rival, and “threatened” after reading the vignette. Responses were given on a 6-point scale ranging from 0 (*not at all*) to 5 (*extremely*). At the end of the study, we also asked participants to indicate their agreement with the statements, “I am interested in hockey” and “I am interested in sport” (see also Leach et al., 2003). The response scale ranged from *strongly disagree* (1) to *strongly agree* (7).

***Experience: pleasure*.** Participants were then asked to indicate the degree to which they felt each of 10 positive emotions (presented with negative emotions to make our purpose less obvious). Responses were given on a 6-point scale ranging from 0 (*not at all*) to 5 (*extremely*). The 10 positive emotions were designed to assess feelings of being generally pleased (i.e., joyful, happy, pleased, jubilant, satisfied), emboldened (i.e., bold, fearless), and triumphant (i.e., triumphant, victorious, proud). A Principal-axis Factor Analysis with maximum likelihood extraction and Oblimin rotation produced these three factors, which were correlated 0.69–0.81. Thus, we constructed scales of feeling generally pleased (α = 0.96), emboldened (α = 0.83), and triumphant (α = 0.93). To capture a particular quality of schadenfreude, we also asked participants whether their “feelings were caused by” “…wanting to get or keep something pleasurable” (1) or “…wanting to get rid of or avoid something painful” (9), based in Roseman et al. (1990).

***Experience: activity*.** Based on Roseman et al. (1990), questions regarding behavioral tendencies asked how much the participant “would feel like” “… jumping up and down” or “…going for it” in the situation they had just read about. Responses were given on a 9-point scale, ranging from 1 (*not at all*) to 9 (*very much so*).

***Experience: elevated phenomenology*.** Participants were next asked how much they would feel the phenomenological experience of elevation that we expect to be most characteristics of gloating: “I would feel ‘10 feet tall’,” “…like I was walking on air,” “…on top of the world.” Responses were given on a 6-point scale ranging from 0 (*not at all*) to 5 (*extremely*). Together these items formed a reliable scale (α = 0.89).

***Expression: gloating*.** Although our method did not rely on participants knowing the meaning of the word gloating, as a face valid test we asked participants if they “would feel like gloating.” Responses were given on a 6-point scale from 0 (*not at all*) to 5 (*extremely*).

***Expression: smiling.*** Based on Roseman et al. (1990), we asked participants if they “…would feel like smiling” or “…would smile” in the situation they had just read about. Responses were given on a 9-point scale, ranging from 1 (*not at all*) to 9 (*very much so*).

***Expression: celebrating*.** To assess their outward expression of celebrating, we asked participants if they “…would feel like celebrating” and “…would feel like holding my head up high.” Responses were given on a 9-point scale, ranging from 1 (*not at all*) to 9 (*very much so*).

***Expression: flaunting*.** Three items assessed the flaunting of pleasure: “…would feel like freely expressing my glee,” “…would feel like flaunting my pleasure,” and “…would feel like boasting.” Responses were given on a 9-point scale, ranging from 1 (*not at all*) to 9 (*very much so*).

***Expression: suppressing.*** We asked participants if they would “…feel like stopping myself visibly smiling” and “...stop myself visibly smiling.” Responses were given on a 9-point scale, ranging from 1 (*not at all*) to 9 (*very much so*). We also asked participants if they would feel “... ashamed for feeling good.” Responses were given on a 6-point scale ranging from 0 (*not at all*) to 5 (*extremely*).

### RESULTS

#### Equivalence checks

The equivalence checks were examined in a series of ANOVAs that treated participants’ sex, schadenfreude vs. gloating vignette, and individual vs. group emotion as factors that could interact. Given the possibility that women and men might differ in their interest in the sport of field hockey we included sex as a factor in these initial analyses.

The feeling of rivalry with the other party was unaffected by the examined factors (all *p*s > 0.13, all $\eta_{p}^{2}$ < 0.020, *M* = 3.63 to 4.15). In addition, hostility toward the rival was consistent across factors (all *p*s > 0.21, all $\eta_{p}^{2}$ < 0.015, *M* = 2.49 to 2.72). Also, participants felt equally “threatened” across emotion conditions, *F*(1,117) = 0.022, *p* = 0.882, $\eta_{p}^{2}$ < 0.001. However, they did feel more threatened in the individual than in the group conditions, *F*(1,117) = 4.75, *p* = 0.031, $\eta_{p}^{2}$ < 0.039. No other effects were significant, all *p* > 0.18, all $\eta_{p}^{2}$ < 0.015.

Participants showed equal interest in sport (*M* = 4.24, SD = 1.88) and in field hockey (*M* = 2.52, SD = 1.63) across conditions, all *p* > 0.18 and all $\eta_{p}^{2}$ < 0.001. As such, this variable was excluded from further analysis. Participants’ sex was also excluded from further analysis because it had little effect here or below.

#### Experience: pleasures

As shown in the top of **Table 4**, participants in the schadenfreude condition attributed their feeling to wanting to avoid pain more than those in the gloating condition. Individual vs. group emotion had no significant main effect, *F*(1,121) = 0.043, *p* = 0.835, $\eta_{p}^{2}$ < 0.001, or interaction effect, *F*(1,121) = 0.800, *p* = 0.373, $\eta_{p}^{2}$ = 0.007.

**Table 4:** The experience of gloating and schadenfreude, Study 2.

	Gloating	Schadenfreude	*F*(df)	*p*	Effect size
	*M* (SE)	*M* (SE)			($\eta_{p}^{2}$)
Want to avoid pain	3.17 (0.251)	4.49 (0.257)	13.60 (1,121)	<0.001	0.101
Pleasures^a^			78.51 (3,119)	<0.001	0.664
General pleasure	4.47 (0.123)	2.28 (0.126)	153.66 (1,121)	<0.001	0.559
Triumphant	4.30 (0.135)	1.50 (0.136)	209.66 (1,121)	<0.001	0.634
Emboldened	2.78 (0.159)	1.47 (0.163)	32.92 (1,121)	<0.001	0.214
Activity^b^			15.80 (2,119)	<0.001	0.210
Jumping up and down	5.94 (0.304)	3.53 (0.309)	31.04 (1,120)	<0.001	0.205
Going for it	6.08 (0.280)	4.71 (0.285)	11.69 (1,120)	<0.001	0.089
Elevated phenomenology^a^			29.53 (3,119)	<0.001	0.427
10 feet tall	3.32 (0.165)	1.89 (0.169)	36.79 (1,121)	<0.001	0.233
Walking on air	2.96 (0.165)	1.36 (0.169)	46.06 (1,121)	<0.001	0.276
On top of the world	3.46 (0.147)	1.47 (0.150)	89.26 (1,121)	<0.001	0.425

*^a^Response scale ranged from 0 *(not at all)* to 5 *(extremely).*^b^Response scale ranged from 1 *(not at all)* to 9 (very much so).*

The three measures of pleasure were analyzed together in a multivariate ANOVA (MANOVA), which showed emotion condition to have a highly significant and large effect (see **Table 4**). Participants reported feeling much more general pleasure, triumphant, and emboldened in the gloating than in the schadenfreude condition. The multivariate effect of Individual vs. Group Emotion was not significant, *F*(3,119) = 1.72, *p* = 0.167, $\eta_{p}^{2}$ = 0.042. The two-way interaction was significant, *F*(3,119) = 6.89, *p* < 0.001, $\eta_{p}^{2}$ = 0.148, although none of the univariate effects was significant (all *p*s > 0.072, $\eta_{p}^{2}$ = 0.026).

#### Experience: activity

The two indicators of activity were analyzed together in a MANOVA, which showed emotion condition to have a highly significant and moderate effect (see **Table 4**). Participants reported that they would feel like “jumping up and down” and “going for it” more in the gloating than in the schadenfreude condition. Individual vs. group emotion did not produce a significant multivariate main effect, *F*(2,119) = 1.15, *p* = 0.321, $\eta_{p}^{2}$ = 0.019, or two-way interaction, *F*(2,119) = 0.557, *p* = 0.575, $\eta_{p}^{2}$ = 0.009.

#### Experience: elevated phenomenology

The three indicators of elevated phenomenology were analyzed together in a MANOVA, which showed emotion condition to have a highly significant and moderate effect (see **Table 4**). Participants reported that they would feel “10 feet tall” “like I was walking on air” and “on top of the world” more in the gloating than the schadenfreude condition. Individual vs. group emotion had a marginally significant multivariate effect, *F*(3,119) = 2.33, *p* = 0.078, $\eta_{p}^{2}$ = 0.055, although none of its univariate effects was significant. The two-way interaction was not significant, *F*(3,119) = 0.704, *p* = 0.552, $\eta_{p}^{2}$ = 0.017.

#### Expression: gloating and smiling

As shown in the first section of **Table 5**, participants imagined “gloating” more in the gloating than in the schadenfreude condition. Neither individual vs. group emotion, *F*(1,120) = 3.49, *p* = 0.064, $\eta_{p}^{2}$ = 0.028, nor the two-way interaction, *F*(1,120) = 0.172, *p* = 0.679, $\eta_{p}^{2}$ = 0.001, was significant.

**Table 5:** The expression of gloating and schadenfreude, Study 2.

	Gloating	Schadenfreude	*F*(df)	*p*	Effect size
	*M* (SE)	*M* (SE)			($\eta_{p}^{2}$)
Gloating^a^	2.02 (0.165)	1.37 (0.170)	7.43 (1,120)	0.007	0.058
Smiling^b^			29.43 (2,120)	<0.001	0.329
Feel like smiling	7.99 (233)	5.86 (0.239)	40.51 (1,121)	<0.001	0.251
Would smile	7.84 (0.246)	5.14 (0.252)	59.34 (1,121)	<0.001	0.329
Celebrating^b^			45.84 (2,119)	<0.001	0.435
Celebrating	7.99 (0.237)	4.88 (0.241)	84.47 (1,120)	<0.001	0.413
Hold head up high	7.51 (0.235)	5.50 (0.239)	35.95 (1,120)	<0.001	0.231
Flaunting^b^			45.84 (3,119)	<0.001	0.154
Freely express glee	6.68 (0.259)	5.01 (0.265)	20.51 (1,121)	<0.001	0.145
Flaunting pleasure	5.94 (0.289)	4.42 (0.296)	13.46 (1,121)	<0.001	0.100
Boasting	6.24 (0.292)	5.04 (0.299)	8.25 (1,121)	0.005	0.064
Suppressing			11.99 (3,119)	<0.001	0.232
Feel like stop smiling^b^	4.20 (0.321)	4.83 (0.329)	1.82 (1,121)	0.180	0.015
Stop smiling^b^	3.06 (0.286)	5.02 (0.293)	22.79 (1,121)	<0.001	0.158
Ashamed^a^	0.84 (0.177)	2.09 (0.181)	24.66 (1,121)	<0.001	0.169

*^a^Response scale ranged from 0 *(not at all)* to 5 (extremely).*

*^b^Response scale ranged from 1 *(not at all)* to 9 (very much so).*

The two questions about the expression of smiling were analyzed together in a MANOVA, which showed emotion condition to have a large and significant effect. Participants reported that they “would feel like smiling” and “would smile” more in the gloating than the schadenfreude condition. Individual vs. group emotion had a small but significant multivariate effect, *F*(2,120) = 4.31, *p* = 0.016, $\eta_{p}^{2}$ = 0.067. Participants reported that they “would smile” more in the group (*M* = 6.95, SE = 0.250) than the individual (*M* = 6.03, SE = 0.248) emotion condition, *F*(2,120) = 6.82, *p* = 0.010, $\eta_{p}^{2}$ = 0.053. The multivariate two-way interaction was not significant, *F*(2,120) = 1.68, *p* = 0.190, $\eta_{p}^{2}$ = 0.027.

#### Expression: celebrating

The two questions about celebrating were analyzed together in a MANOVA, in which emotion had a large and significant effect (see **Table 5**). Participants “would feel like celebrating” and “would feel like holding my head up high” more in the gloating than in the schadenfreude condition. Individual vs. group emotion had a marginal multivariate effect, *F*(2,119) = 3.02, *p* = 0.052, $\eta_{p}^{2}$ = 0.048. The two-way interaction was not significant, *F*(2,119) = 1.55, *p* = 0.216, $\eta_{p}^{2}$ = 0.025.

#### Expression: flaunting

The three questions about flaunting one’s pleasure were analyzed together in a MANOVA, in which emotion had a significant and moderate-sized effect (see **Table 5**). Participants “would feel like freely expressing my glee,” “would feel like flaunting my pleasure,” and “would feel like boasting” more in the gloating than in the schadenfreude condition. Individual vs. group emotion had a small, significant multivariate effect, *F*(3,119) = 3.08, *p* = 0.030, $\eta_{p}^{2}$ = 0.072. Participants said that they would more freely express their glee in the group (*M* = 6.32, SE = 0.263) than in the individual (*M* = 5.37, SE = 0.261) condition, *F*(1,121) = 6.64, *p* = 0.011, $\eta_{p}^{2}$ = 0.052. The two-way interaction was not significant, *F*(3,119) = 0.094, *p* = 0.963, $\eta_{p}^{2}$ = 0.002.

#### Expression: suppressing

The three questions about suppressing one’s pleasure were analyzed together in a MANOVA, in which emotion had a significant medium-sized effect (see **Table 5**). Participants “would feel that I had to stop myself visibly smiling,” feel “…ashamed for feeling good” and “would stop myself visibly smiling” more in the schadenfreude than the gloating condition. Individual vs. group emotion was also significant, *F*(3,119) = 6.35, *p* < 0.001, $\eta_{p}^{2}$ = 0.138, as participants expected to stop smiling and to feel ashamed more in the individual than in the group emotion condition (both *p* < 0.001, $\eta_{p}^{2}$ > 0.08). The two-way interaction was not significant, *F*(3,119) = 0.880, *p* = 0.454, $\eta_{p}^{2}$ = 0.022.

### DISCUSSION

Importantly, equivalence checks showed that participants were equally interested in sport in general, and field hockey in particular, across the experimental conditions. In addition, participants’ sense of rivalry, their hostility, and their feeling threatened by the events described, were equivalent across experimental conditions. Thus, there was little difference in what participants had “at stake” in the schadenfreude and gloating situations, or in the individual and group situations. This eliminates an obvious alternative explanation of our findings, namely that the events were viewed differently in other important respects to those manipulated.

Despite the fact that the schadenfreude and gloating conditions were of similar relevance to participants, they expected to experience these two situations quite differently. Those who were led to imagine that they (or their university team) had passively observed a rival fail anticipated feeling much less pleasure than those who imagined outdoing the rival themselves. Those in the schadenfreude condition also expected to feel less of the empowered pleasure assessed with feeling triumphant and emboldened. Consistent with this, schadenfreude was expected to be a less active experience than gloating. And, gloating was seen as involving much more of the embodied experience of elevation than schadenfreude. Thus, gloating was thought to make one feel “on top of the world.” In sum, Study 2 corroborated and extended Study 1 by showing that gloating and schadenfreude situations are characterized by different experiences of pleasure. As Nietzsche (1887/1967, p. 67) argued, “to see others suffer does one good, to make others suffer even more.”

Participants also reported quite dramatic differences in how they expected to express their pleasure in gloating and schadenfreude. We expected that defeating a rival oneself would lead to outright gloating and much more smiling and celebrating. Indeed, participants expected to flaunt their pleasure much more in the case of gloating than schadenfreude. Overall, the expression of pleasure at simply observing a rival’s failure was expected to be moderate at best. In fact, participants actually expected to suppress their visible smiling and to feel ashamed about feeling the pleasure of schadenfreude. This is consistent with our suggestion that schadenfreude is seen as being of questionable legitimacy and is thus furtive in nature (see Leach et al., 2003).

There were again few differences between the individual and group examples of gloating and schadenfreude. Where there were differences, they tended to be small. One trend was for group emotions to be expressed more freely and for individual emotions to be slightly more furtive. This probably reflects the fact that group-based emotions offer the potential for a relatively consensual appraisal of events, whereby fellow group members can be expected to share and thereby validate the emotional experience (for discussions, see Tiedens and Leach, 2004; Parkinson et al., 2005).

## GENERAL DISCUSSION

Together these studies offer a multi-method examination of the distinctions between two pleasures at other’s adversity – schadenfreude and gloating. The emotion recall and vignette methodologies produced similar results. In both cases we avoided reference to emotion words in our methods. Thus, we were able to define the pleasures of interest more precisely, without relying on participants’ potentially idiosyncratic understanding of emotion words. Across both studies there were few differences between the individual and group examples of gloating and schadenfreude. Group-based emotions seemed to increase expression slightly, likely because individuals can presume that such emotions are shared and thus socially validated (for discussions, see Tiedens and Leach, 2004; Parkinson et al., 2005). Although there are ways in which individual and group-based emotion may differ, the appraisals, phenomenology, and motivation that we examined here should be similar if the precipitating events are similarly self-relevant (Iyer and Leach, 2008).

It is worth acknowledging possible limitations of our approach. The most obvious of these is our reliance on self-report, a method with well-known drawbacks. Nevertheless, self-report seemed to be the most appropriate way to access the detailed and complex dimensions (i.e., appraisals, feeling states, and action tendencies) that define complex emotions such as schadenfreude and gloating. Although alternative methodologies that capture emotional experience less explicitly (e.g., EEG, fMRI, facial expressions) might be able to provide important complementary evidence, the differences we observe between schadenfreude and gloating represent an important first step in establishing the distinctions between these malicious pleasures. Indeed, it is not clear how many of these distinctions could be studied with methods that do not rely on the conscious reporting of the subjective meaning of these emotions.

A second possible limitation is our use of vignettes in Study 2. Such methodologies have been criticized on the grounds that they present participants with hypothetical scenarios and thereby elicit responses guided by lay theories (Parkinson and Manstead, 1993). However, it is important to note that Study 1 used personally experienced rather than hypothetical events, yet yielded similar results to Study 2. This echoes the evidence that vignettes designed to study emotional experience can generate results that parallel those found with non-vignette methodologies (Robinson and Clore, 2001). It likely helped that the vignettes used in Study 2 were designed to mimic real-life individual and group competition relevant to the participants.

### EMOTION AS RELATIONAL

People who express emotion, like those who study emotion, share a rich and varied vocabulary for dysphoric feelings. Our language for euphoric feelings is more limited (Averill, 1980; de Rivera et al., 1989; more generally, see Frijda, 1986; Shaver et al., 1987; Ortony et al., 1988; Lazarus, 1991). Yet, it is evident that all pleasures are not the same. The elation at winning the lottery is different from the pride in seeing a daughter graduate or the joy in watching the sun set. Although pleasures at bad things that happen to other people have a certain malice in common, they too are different from one another. The conflation of schadenfreude and gloating in academic and popular discussion masks the ways in which these two pleasures differ in terms of situational features, appraisals, experience, and expression. Just as Nietzsche suggested, schadenfreude is a modest, furtive, guilty pleasure that does little to empower those who experience it. Gloating is a very different pleasure. It is about a direct and active outperformance of another party who is then made to witness one’s pleasure at their defeat. Gloating is not only a greater experience of pleasure. In contrast to schadenfreude, gloating is experienced as a physical invigoration and elevation of the body. People beam as they “walk on air,” elevated above their defeated rivals. A little smile, and a quiet satisfaction, is all that people seem to get from schadenfreude.

The many distinctions observed between schadenfreude and gloating illustrate the ways in which emotional experience and expression is situated in social relations. Despite being close cousins within the broader family of pleasures, and siblings within the family of pleasures at other’s adversity, gloating and schadenfreude are very different ways of relating to the social world. Although taking pleasure in another’s adversity necessarily positions one against the other, the pleasure of schadenfreude was not flaunted. In fact, it was suppressed to some degree. As such, schadenfreude seems unlikely to lead to more direct derogation or more active mistreatment of the other party (see Leach et al., 2003; Leach and Spears, 2009). What is gained in schadenfreude is a modest psychological boost for the self (Leach and Spears, 2009). In contrast, gloating is a more active and direct opposition to the other party. The pleasure of gloating was not only experienced more intensely, it was expressed more intently. These emboldened expressions of presumed superiority seem much more likely to fuel further antagonism. Gloating may even encourage the defeated rival to seek revenge or retribution for the indignity they have been made to suffer. As such, gloating may present a greater risk to social relations than schadenfreude because the experience and expression of gloating empower more, and more direct, antagonism. By parsing the malicious pleasures of gloating and schadenfreude, we have taken a first step toward understanding how these two emotions are likely to affect the (individual or group) relations within which they are embedded.

## Conflict of Interest Statement

The authors declare that the research was conducted in the absence of any commercial or financial relationships that could be construed as a potential conflict of interest.

## Footnotes

1 One-hundred and twenty-one students (103 women, 18 men) were originally recruited. They identified as English (60), British (28), Welsh (14), Irish (2), Scottish (1), or “other” (16). Out of concern that participants might not report appropriate narratives in the more complicated case of schadenfreude, we assigned 40 participants to this condition. Two independent coders examined whether the schadenfreude narratives conformed to instructions. We were most concerned about the schadenfreude narratives actually being examples of gloating. Thus, coders identified ostensible schadenfreude narratives that referred to instances of directly outperforming another party. The coders agreed in 90% (i.e., 72) of the 80 cases. Disagreements were settled by discussion. The coders found that 12 of the 40 participants in the schadenfreude conditions reported pleasure at directly outdoing a rival in both their individual and group narrative. Such events are examples of gloating or pride, rather than schadenfreude. Indeed, these 12 participants tended to describe their feelings as “smug,” “proud,” or “superior.” For example, when asked for an example of individual schadenfreude, a participant reported an event involving an “intelligent” classmate whose parents “would always try to brag about her and compare her to me.” The participant reported that the two girls got “almost identical” grades, except for in Spanish where the participant received an A and her rival received a C. She summarized her feeling as “satisfaction/smugness.” This is an example of gloating rather than schadenfreude in our view. Eliminating the 12 participants who failed to produce any narrative that conformed to schadenfreude left 28 participants in this condition. Of the 56 (individual + group) narratives that they produced, 16 involved outdoing a rival. Thus, only 71% of these narratives are “pure” cases of schadenfreude. However, we chose to retain all 56 narratives in the schadenfreude condition to keep cell sizes near equal. It is important to note that this approach makes our comparison of the schadenfreude conditions to the others a more conservative test of our hypotheses.

2 These Chi-square tests use Yates’s correction for continuity to improve the accuracy of tests that include cells with small or zero values (see Preacher, 2001).
